# Therapeutic Aspects of Carbon Monoxide in Cardiovascular Disease

**DOI:** 10.3390/ijms19082381

**Published:** 2018-08-13

**Authors:** Hyuk-Hoon Kim, Sangchun Choi

**Affiliations:** Department of Emergency Medicine, Ajou University School of Medicine, Suwon 16499, Korea; hyukhoon82@gmail.com

**Keywords:** carbon monoxide, therapeutics, cardiovascular diseases, adverse effects

## Abstract

Carbon monoxide (CO) is being increasingly recognized as a potential therapeutic with important signaling functions in various diseases. Carbon monoxide-releasing molecules (CORMs) show anti-apoptotic, anti-inflammatory, and anti-oxidant effects on the tissues of organisms, thus contributing to tissue homeostasis. An increase in reactive oxygen species production from the mitochondria after exposure to CO is also considered one of the underlying mechanisms of cardioprotection, although mitochondrial inhibition is the main toxic mechanism of CO poisoning. This review highlights the mechanism of the biological effects of CO and its potential application as a therapeutic in clinical settings, including in cardiovascular diseases. This review also discusses the obstacles and limitations of using exogenous CO or CORMs as a therapeutic option, with respect to acute CO poisoning.

## 1. Introduction

Carbon monoxide is one of the most harmful substances that have deleterious effects on eukaryotic organisms and is a common cause of morbidity and mortality resulting from poisoning in humans [1,2]. Also known as a silent killer, CO is one of the main causative substances of poisoning-related deaths in the United States and European Union [3,4]. Until recently, clinicians specializing in toxicology have concentrated on decreasing and preventing CO poisoning-related deaths and complications.

The symptoms and signs of CO poisoning and CO poisoning-related deaths result from tissue hypoxia due to the high affinity of CO for hemoglobin, which is approximately 210- to 250-times higher than the affinity of oxygen to hemoglobin at normal atmospheric pressure [5,6]. The binding of CO to heme moieties in hemoglobin causes an allosteric conformational change in hemoglobin, forming carboxyhemoglobin (COHb), such that it cannot release oxygen from other oxygen binding heme moieties to peripheral tissues [7,8]. Hypoxia, induced by the blockade of oxygen release from hemoglobin, disrupts the oxidative phosphorylation of mitochondria in cells, which results in the fatal inhibition of ATP synthase and accumulation of superoxide [9,10,11]. Therefore, the first-line of treatment in a CO-poisoned patient is an abundant oxygen supply to increase competition with CO in binding to hemoglobin and re-releasing oxygen to tissues. Thus, hyperbaric oxygen therapy has been established as a recommended therapeutic method in patients who have been severely poisoned with CO [12,13,14].

However, CO is endogenously synthesized by heme-oxygenase, which is inducible by various circumstances or is constitutively expressed in several organs. CO has been recognized as one of the gasotransmitters or gaseous messengers that regulates metabolism in humans [15,16,17,18]. Using the newly identified, biologically beneficial functions of CO, several clinical trials have been performed which analyze the effect of exogenous CO, whether inhalation-based or CO-releasing molecule-based, to test the preconditioning of human mitochondrial biogenesis in aortic valve surgery patients (NCT01727167), to suppress lung inflammatory responses following endotoxin instillation (NCT00094406), to modify chronic inflammation in chronic obstructive pulmonary disease (NCT00122694), to decrease the post-operative ileus after colon resection (NCT01050712), to treat sepsis-induced acute respiratory distress syndrome (NCT02425579), and to improve organ tolerability in kidney transplantation (NCT00531856).

**Figure 1:**
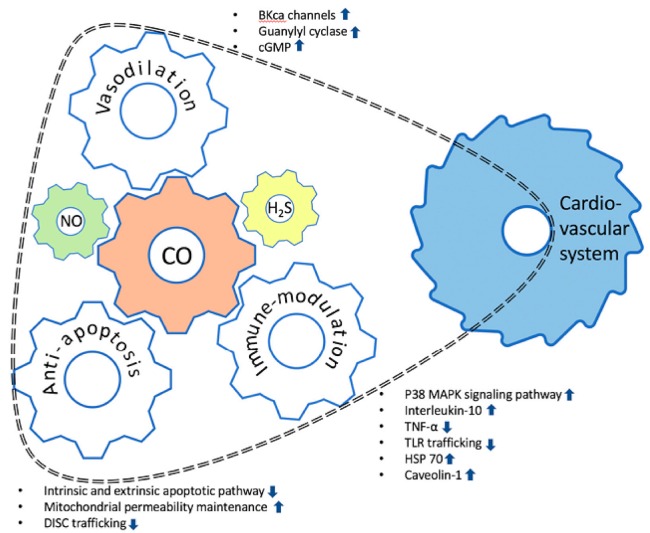
Schematic diagram of the effects of carbon monoxide on the cardiovascular system. Carbon monoxide (CO) can influence the cardiovascular system through (1) the vasodilatory effect, (2) anti-apoptotic activity, and (3) immune-modulation mechanisms. The roles of CO in the cardiovascular system may be executed in conjunction with other gasotransmitters, such as nitric oxide (NO) and hydrogen sulfide (H_2_S). The possibility of action on the cardiovascular system is represented by the dotted line. The upward pointing arrow and the downward pointing arrow mean activation and inhibition for each pathway, respectively.

The positive effects of CO whichcould be clinically applicable, especially for cardiovascular disease, are as follows: (1) vasorelaxing activity via the regulation of vascular smooth muscle tone; (2) anti-inflammatory effects through the regulation of the expression and release of various cytokines; and (3) anti-apoptotic reaction by inhibiting the cellular apoptotic pathway in mitochondria (Figure 1) [19,20,21]. Cardiovascular disease might be the choice with the most potential for the therapeutic use of CO. However, many mechanisms underlying the clinical effects of CO on the cardiac tissue still need to be elucidated. This review can be beneficial in terms of the identification and evaluation of the clinical applicability and the obstacles in the clinical application to cardiovascular disease. This review highlights the underlying mechanisms of the biological effects of CO and describes the applications of CO in clinical settings, including in cardiovascular diseases. Further, this review also discusses the limitations of using exogenously supplied CO as a therapeutic tool, particularly in the context of CO poisoning.

## 2. Vessel Relaxing Activity

Carbon monoxide plays important functions in the regulation of vascular tone, mainly by activating the large-conductance calcium-activated potassium channel (BK_Ca_ channel) and partly by activating soluble guanylyl cyclase in smooth muscle cells. CO dilates the arteries and arterioles by binding to tetrameric voltage-gated so-called “big potassium” channels that conduct large amounts of potassium ions (K^+^) across the cell membrane in smooth muscle cells of the blood vessel [22]. The BK_Ca_ channels can be activated or opened by electrical means or by increasing the calcium concentration in the cell, and their function is to repolarize the membrane potential by allowing potassium to flow outward [23]. The BK_Ca_ channels are composed of a α-subunit that forms a pore and an auxiliary β_1_-subunit, which raises the sensitivity of the channel for Ca^2+^. CO sensitizes the BK_Ca_ channels and regulates channel activity in arterial smooth muscle cells to maintain the intracellular Ca^2+^ levels within the micromolar concentration [24,25].

To activate BK_Ca_, local Ca^2+^ transients, termed “Ca^2+^ sparks”, are needed. These Ca^2+^ sparks generate the necessary micromolar [Ca^2+^] at the intracellular surface of the BK_Ca_ channel by activating the sarcoplasmic reticulum ryanodine-sensitive Ca^2+^ release channel (RyR channel) [26,27]. Some portion of the BK_Ca_ channels may respond to and be activated by a single Ca^2+^ spark, and this leads to a transient outward K^+^ flow through the BK_Ca_ channel. In the arterial wall, transient BK_Ca_ currents hyperpolarize the membrane potential and reduce the activities of voltage-dependent Ca^2+^ channel located in the cellular membrane. This leads to a decrease in the global intracellular [Ca^2+^] concentration, finally resulting in the vasorelaxation response in the blood vessel.

The vasorelaxation activity of CO is mediated by binding with the α-subunit of BK_Ca_ channels and activation of the channels. Cellular heme, which is produced in the reduced state, binds to the heme-binding domain (Cys-Lys-Ala-Cys-His) of the α-subunit, located between amino acids 612 and 616. Thus, BK_Ca_ channels exist as functional heme proteins, and this binding of reduced heme inhibits the BK_Ca_ channels [28,29]. The binding of CO to BK_Ca_ channel-bound reduced heme alters the association of heme with the channel, leading to an enhancement in activity [30]. Thus, BK_Ca_ channel-bound heme is a receptor for CO, and CO binding increases the Ca^2+^ sensitivity of the BK_Ca_ channel [31]. By increasing the Ca^2+^ sensitivity of the BK_Ca_ channel, CO increases the fractional coupling of BK_Ca_ channels activated by Ca^2+^ sparks [30,31]. Though a significant proportion of Ca^2+^ sparks—up to about 30%—does not evoke a transient K^+^ current in humans, 100% of Ca^2+^ sparks evoke a transient K^+^ current following the elevation of Ca^2+^ sensitivity of BK_Ca_ channels by CO [32,33]. CO also increases the coupling frequency of the BK_Ca_ channel by increasing the frequency of Ca^2+^ sparks through direct activation of RyR channels and leads to an increase in the transient BK_Ca_ current frequency [34,35]. Even though there are some differences in response to CO, the vasodilatory effect of CO can be explained by these combined effects, which increase both the fractional and effective coupling of BK_Ca_ channels to Ca^2+^ sparks.

Vasorelaxation by CO-induced cell signaling has been proposed to occur via the activation of soluble guanylyl cyclase, although CO is far less effective at activating guanylyl cyclase than nitric oxide (NO) [36,37]. The elevation of cyclic GMP in smooth muscle cells leads to the activation of cGMP-dependent protein kinase G (PKG). Activated PKG induces the reuptake of Ca^2+^ by the sarcoplasmic reticulum and results in the decrease of intracellular [Ca^2+^] concentration and the relaxation of smooth muscles [38]. Additionally, activated PKG phosphorylates RyR channels in the sarcoplasmic reticulum, and this phosphorylation also increases Ca^2+^ sparks, which are related to vasorelaxation [39]. Although further investigation is necessary to qualify this vasodilatory effect of CO, this effect could be applied in the medical field to control vascular tone. First, the regulation of vascular tonicity could be used to regulate the blood pressure in patients with essential hypertension. Further, CO may be one of the solutions in ischemic injury resulting from the circulatory insufficiency caused by unwanted vascular constriction. For example, vasodilatory action could be helpful in acute coronary syndrome, especially in variant angina, by dilating coronary arteries. CO may also increase the renal blood flow, glomerular filtration rate and decrease the renal vascular resistance in acute kidney injury by dilating the renal artery and arteriole. Moreover, in pulmonary hypertension with or without heart failure, CO could be a potential solution to enable the release of the pressure in pulmonary and systemic vasculature.

## 3. Anti-Apoptotic Property

Apoptosis can be initiated through one of two pathways: the intrinsic pathway, which is activated by intracellular signals generated when cells are stressed and depends on the release of proteins from the mitochondrial intermembrane space; and the extrinsic pathway, which is activated by extracellular ligands binding to cell-surface death receptors, leading to the formation of the death-inducing signaling complex (DISC) [40]. The cytoprotective effect by CO is associated with both cellular apoptotic pathways [41].

Apoptosis initiated by the intrinsic pathway is related to mitochondrial membrane permeability [11]. Mitochondrial membrane permeabilization leads to the process of irreversible programed cell death via the following mechanisms: (1) the extinction of mitochondrial transmembrane potential; (2) the uncoupling of oxidative phosphorylation; (3) excessive production of reactive oxygen species; (4) cessation of ATP synthesis; and (5) the release of pro-apoptotic proteins [42].

The main mechanism by which CO mediates the anti-apoptotic action in the intrinsic pathway is the prevention of the association of Bid and Bax, which are pro-apoptotic members of the Bcl-2 family at the outer mitochondrial membrane. CO inhibits the expression and mitochondrial translocation of Bax [41]. CO also prevents the activation of Bid by inhibiting the activity of caspase-8, which activates Bid by cleavage to truncated Bid (tBid) [43]. The activated tBid is a direct activator and inducer of Bax, causing a conformational change that enables Bax oligomerization and insertion into the outer mitochondrial membrane [44,45]. Oligomerized Bax is enabled by tBid, not only through protein–protein interactions, but also by protein–lipid interactions, to form lipid pore-type structures in the outer mitochondrial membrane. Then, oligomerized Bax facilitates the release of cytochrome c and other pro-apoptotic molecules from the mitochondrial intermembrane space [46,47,48,49].

With respect to the extrinsic apoptotic pathway, CO inhibits the formation of a death-inducing signal complex (DISC) at the plasma membrane by preventing DISC trafficking from the Golgi apparatus to the plasma membrane. The extrinsic apoptotic pathway is initiated by a death ligand, such as the Fas ligand (FasL) which interacts with its cell surface receptor (i.e., Fas) and forms a death-inducing signal complex (DISC) [50]. The activation of Fas induces its oligomerization and the rapid recruitment of FADD (Fas-associated death domain protein) and caspase-8, which constitute the DISC. Autoproteolytic generation of caspase-8 from pro-caspase-8 occurs within the DISC [51]. Although the precise mechanisms underlying DISC formation and the translocations of Fas, FADD, and caspase-8 are still uncharacterized, the formation of DISC occurs in the Golgi apparatus and then DISC translocates to the plasma membrane [52,53]. DISC formation in the Golgi complex is necessary in the apoptotic pathway, while its location in the plasma membrane is essential for caspase-8 activation [41,52]. Activated caspase-8 subsequently cleaves Bid into tBid, which translocates from the cytosol to the mitochondrial membrane, where it assists in the activation of Bax, the main molecule in the intrinsic pathway [54]. In this extrinsic pathway signaling, CO performs a cytoprotective role against death receptor-mediated apoptosis by diminishing DISC formation in the Golgi complex and plasma membrane and by inhibiting the associated activation of caspase-8 and Bid. Other mechanisms have been suggested for the role of CO in the extrinsic pathway, such as the activation of the p38 mitogen-activated protein kinase signaling pathway and NK-kB upregulation; the resultant FADD-like ICE inhibitory protein activation inhibits the TNF-α/Act-D induced caspases-8 cleavage [55,56].

The anti-apoptotic effects of CO could be clinically applied in areas where the improvement of cell survival is desired to protect against acute stress or chronic destructive changes. For example, ischemic stroke and acute coronary syndrome are representative diseases where injury by ischemia is caused by circulation insufficiency. Even though in such cases the definitive treatment is the reperfusion by re-vascularization, the restoration of blood flow to injured area also causes additional injury, the so-called ischemia-reperfusion injury (IRI). In such conditions, the cytoprotective effect of the anti-apoptotic property of CO may reduce tissue injury by IRI, as well as by initial ischemia. In addition, it may be possible to delay the progression of neurodegenerative diseases, such as Alzheimer’s, Parkinson’s, and Lou Gehrig’s disease which are caused by undesired chronic degeneration, and to slow the progression of heart failure caused by chronic myocardial damage and resultant myocyte apoptosis.

## 4. Inflammation Modulatory Effects

CO can participate in several aspects of the inflammatory pathway and result in anti-inflammatory effects by the suppression of the immune reaction. One of the possible mechanisms for the CO-mediated inhibition of inflammation is via the suppression of the production and release of TNF-α in lipopolysaccharide (LPS)-stimulated macrophages [57]. In this mechanism, CO increases LPS-induced expression of the anti-inflammatory cytokine interleukin-10 (IL-10). IL-10 plays an important role in the anti-inflammatory reaction by inhibiting TNF-α production via the activation of p38 MAPK signaling pathway [58]. The increase in IL-10 released from macrophages in response to CO activation promotes the expression of heme oxygenase-1, which produces CO from heme molecules, indicating that CO and IL-10 form a positive feedback circuit reinforcing their anti-inflammatory potential via the upregulation of their expressions. 

Other potential mechanisms underlying the anti-inflammatory effects of CO involve the inhibition of toll-like receptor trafficking to membrane lipid rafts [59]. Toll-like receptors (TLRs) are expressed on sentinel cells such as macrophages and dendritic cells. The recognition of molecules derived from pathogens by TLRs activates signaling pathways that induce the expression of proinflammatory genes. The trafficking of TLRs to the lipid rafts in response to pathogens is dependent on reactive oxygen species (ROS) [59,60]. CO modulates the TLR signaling pathway by inhibiting the translocation of TLR to lipid rafts through the suppression of NADPH oxidase-dependent ROS generation [59].

In addition, with respect to the activation of the P38 MAPK signaling pathway, CO increases the expression of heat shock protein (HSP) 70, which is the major inducible HSP synthesized by temperature-induced adaptive genetic change, and caveolin-1, which serves as the principle structural component of plasma membrane caveolae and potentially regulates many downstream signaling processes that originate in the membrane. The elevated expressions of these molecules have been shown to exhibit the anti-inflammatory effects [61,62,63,64].

Inflammation is a protective response for the elimination of harmful pathogens. However, dysregulated inflammatory reactions are deleterious, such as in sepsis or autoimmune diseases. Sepsis is a life-threatening organ dysfunction caused by a disproportionate host response to infection, and CO could be applied to modulate the dysregulated immune response [65]. Autoimmune diseases, including ankylosing spondylitis, psoriasis, psoriatic arthritis, Behcet’s disease, arthritis, inflammatory bowel disease (IBD), and allergy, are conditions arising from an abnormal immune response to the body’s own components or molecules, and these unwanted and excessive immune reactions could also regulated by CO administration. In addition, both acute and chronic graft-versus-host disease that occurs after organ transplantation could benefit from the anti-inflammatory effects of CO.

## 5. Other Favorable Effects

In addition to the above, the favorable effects of CO may include anti-proliferative effects on smooth muscle cell and the modulation of mitochondrial respiration related with uncoupling and preconditioning [9,66]. CO directly down-regulates expression of cyclin D1, a key regulator of G1 progression, and upregulates expression of p21^Cip1^, a potent inhibitor against cell cycle progression, through the ERK1/2 MAPK pathway, which results in arresting cells at the G0/G1 phase of the cell cycle [67]. In addition, CO inhibits the changes of the smooth muscle cell from the quiescent contractile state to the active synthetic state by suppressing the action of growth factors or cytokines that cause cell proliferation [68,69,70]. Through these mechanisms, CO could exhibit the anti-proliferative effect on smooth muscle cell located in vascular system.

Furthermore, CO could promote the uncoupling of mitochondrial respiration and modulate the production of reactive oxygen species (ROS). In mitochondrial oxidative phosphorylation, 1–3% of the consumed oxygen is incompletely reduced to anion superoxide, which is the primary ROS produced by the electron transport chain, and this generation of a moderate free radical can lead to a more reactive or secondary ROS derivative, even under physiological conditions [11,71]. Under pathological conditions, the reversion of electron flow might result in persistent and augmented generation of ROS; thus, mild mitochondrial uncoupling is an inherent cellular mechanism to limit excessive oxidative stress [9]. The uncoupling of mitochondrial respiration could be activated by CO through the stimulation of the activity of uncoupling proteins and/or the ATP/ADP translocator, which play an important role in uncoupling reactions at a low level of CO [66]. This activation of uncoupling in mitochondrial respiration may prevent the excessive generation of ROS and consequently indicate the anti-oxidant properties of CO.

Although it could be contrary to anti-oxidant properties, CO may partially and/or contrarily inhibit the mitochondrial electron transport chain and lead to an accumulation of electrons, which facilitates anion superoxide [11]. The mild oxidative stress on mitochondria, so-called preconditioning, ultimately enhances the energy production in mitochondria by accelerating the oxidative phosphorylation and improving the mitochondrial respiration [9]. By these preconditioning effects on mitochondrial biogenesis, CO could have a positive role in cellular survival.

## 6. The Therapeutic Potential of CO for Cardiovascular Diseases

There might be possibilities for the immediate application of the positive effects of CO in clinical practice, and the struggles in the treatment of cardiovascular diseases could be alleviated by the therapeutic use of CO. First, due to its modulation of vascular tone, CO could be an additional therapeutic option in patients whose hypertension is not adequately controlled by existing blood pressure-lowering agents, such as calcium channel blockers or angiotensin receptor blockers. The cytoprotective effect resulting from the anti-apoptotic property of CO might contribute to the reduction in hypoxia-induced cardiac muscle cell death during insufficiency in coronary artery circulation. Thus, CO might be used as an adjunctive therapy in acute myocardial infarction. In addition, cell death in the heart, caused by reperfusion injury after percutaneous coronary intervention or thrombolysis, could be decreased by the anti-apoptotic activity of CO. When the heart is subjected to ischemic stress or exposed to sustained enhancement in intraventricular pressure, all nucleated cells, including cardiomyocytes, produce pro-inflammatory cytokines as a response to injury. For example, during MI, myocarditis, or heart failure, both tumor necrosis factor α (TNF-α) and interleukin 6 (IL-6) levels increase in proportion to the severity and duration of congestive heart failure (CHF). This cytokine release may trigger a cascade of events leading to myocardial structural alterations, further deteriorating the clinical picture of CHF. The modulation of inflammation and vasoactive responses might be beneficial to CHF. Furthermore, in heart transplantation, the organ survival rate could be increased by decreasing the transplant rejection response by modulating the immune reaction of the recipient. In addition, through the anti-proliferative effect on smooth muscle cells, the formation of atherosclerosis that causes circulatory insufficiency in the coronary artery could be inhibited by CO. In the process of atherosclerosis formation, smooth muscle cell proliferation and monocyte recruitment are essential steps after endothelium injury [72]. CO inhibits the proliferation of smooth muscle cells directly and indirectly, thereby inhibiting the formation of atherosclerosis [73].

**Figure 2:**
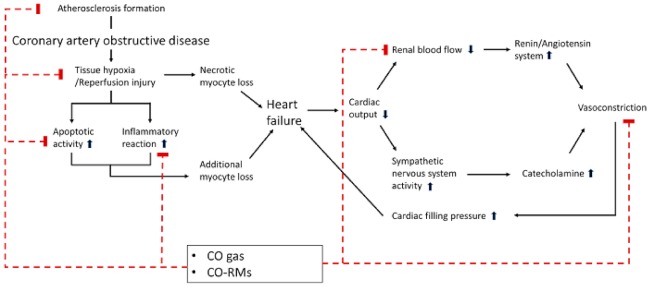
Mechanisms of the favorable effects of carbon monoxide in cardiovascular diseases. Carbon monoxide (CO) can exhibit inhibitory effects on the initiation and progression of cardiovascular disease. (1) CO could suppress the formation of atherosclerosis in the coronary artery and prevent the initiation of coronary artery obstructive disease by the inhibition of the proliferation of smooth muscle cells in the coronary artery. (2) Through anti-apoptotic and anti-inflammatory properties, CO could diminish the loss of myocyte following acute coronary syndrome. (3) The vasodilatory effects of CO can prevent the vicious cycle of heart failure by inhibiting vasoconstriction as a result of the activated renin/angiotensin system and the sympathetic nervous system to compensate for low cardiac output and systemic hypo-perfusion in heart failure. The upward pointing arrow and the downward pointing arrow mean activation and inhibition for each pathway, respectively.

Thus, the above-mentioned clinical applications of CO can result in positive effects, particularly in the context of cardiovascular diseases. So far, the administration of CO in expectation of these beneficial effects in the cardiovascular system has been executed by a delivery device for inhaled gas or by a drug with CO releasing molecules (CORMs) [19]. CORMs are molecules that can release CO under certain biological conditions, such as thermal activation or hydrolysis in biological buffers, and CORMs as a type of prodrug designed for disease-specific targeting could provide the therapeutic action needed for treating cardiovascular disease (Figure 2) [74,75,76].

## 7. Obstacles Against CO Application

It is astounding that CO, which is considered completely toxic, has emerged as a new therapeutic substance. However, in medical practices, many clinicians are currently concentrating on reducing the morbidity and mortality of patients suffering from carbon monoxide poisoning, i.e., to reduce the toxic effects of carbon monoxide [77]. Studies regarding the complications and sequelae of CO poisoning, such as delayed neurological sequelae (DNS), permanent brain injury, and cardiovascular abnormalities in CO poisoning, are still being actively conducted [78,79]. Therefore, the application of CO to treat clinical disease might be counter-intuitive and lead to hesitancy in the application of CO as a remedy for patients. In addition, although many experimental and clinical studies have demonstrated the vasorelaxatory, anti-apoptotic, and anti-inflammatory properties of CO, there are still limitations in the clinical application of CO.

One of the limitations of the medical application of CO is the lack of a precise monitoring method for CO in tissues or organs. Recently, several modalities, such as inhalers or CO releasing molecules (CORMs), for the delivery of CO to tissues or organs in lower concentrations have been introduced. The elevation of COHb in blood to 15% by these novel modalities was tolerable with no documented severe adverse events. However, reliable and accurate monitoring techniques for checking the precise concentration of CO in tissues and organs are still unavailable [80,81]. Further, research on the interaction between CO and existing drugs and the effect of these interactions on the activity of CO in tissues or organs is still lacking. The therapeutic effect of CO seems dose-dependent. Without accurate measurement of the concentration of CO in tissues, using CO in a homogeneous manner without sufficient consideration of the various situations in which the concentration of CO may change could be detrimental to the safety of the patient.

The positive effects of CO, which are shown in experimental studies, are different depending on the cell or tissue in question, and a clear mechanism for this difference still needs to be elucidated. While several studies have shown the cardioprotective effect of CO or CORMs, these have not yet reached clinical practice. For example, the responses of blood vessels to CO were different depending on the location of the blood vessels, and the role of the CO-activated P38 MAPK pathway in modulating the inflammatory responses were different depending on the cell type [58,82]. Moreover, these variable responses to treatment in different tissues and organs could be accompanied by the risk of unexpected side effects. Therefore, it is still early to consider CO as a therapeutic, since it has only been proven to have a positive effect on specific individual organs.

In addition, the positive effects shown in various studies are mostly based on short-term results from experiments ranging from a few minutes to several days. Even if exposure to CO at a low concentration for a short time is considered harmless, there may be concerns about unexpected complications resulting from long-term exposure. These concerns must be addressed by confirming that long-term exposure to CO will also have positive effects and that no organs are particularly vulnerable to long-term exposure.

## 8. Conclusion and Suggestions for Future Research

In conclusion, there are still various obstacles to applying CO or CORMs in cardiovascular disease as therapeutic agents, primarily due to their dose-dependent dual nature. There are further challenges to the use of CO as a novel therapeutic agent, rather than as a toxic substance, which need to be resolved. First, ensuring the safety of the patient during therapeutic exogenous administration of CO or CORMs is critical. As with CORMs, methylene chloride is released slowly from the tissues, resulting in the accumulation of CO for a prolonged period, which causes acute CO intoxication [83]. Therefore, when CO and CORMs are used for therapeutic purposes, continuous monitoring of the CO concentration and the establishment of a precise therapeutic range for safe treatment is crucial; extensive future research will be needed in order to address these concerns.

Further, considering the characteristics of recent CORMs, it is necessary to develop a specific-purpose formulation that exhibits the therapeutic effect only in the target organ, in order to minimize the occurrence of adverse effects. Moreover, it is probable that the therapeutic effects of CO in the body occur due to the interactions of CO with other gasotransmitters, such as nitric oxide (NO) and hydrogen sulfide (H_2_S), rather than being due to the action of CO alone. Therefore, further studies on the mutual interactions between gasotransmitters in therapeutic concentrations are required. Finally, the development of an early and precise predictor for complications such as DNS or CO-induced cardiomyopathy, one of the most notorious complications related with CO poisoning, is also necessary for the application of CO or CORMs in the clinical field.

Thus, the most important problem related with the clinical use of CO or CORMs is the occurrence of serious adverse effects, such as DNS or CO-induced cardiomyopathy. Therefore, research on the identification and characterization of early predictors for DNS or CO-induced cardiomyopathy are essential. It is vital that such studies be conducted in parallel with those on the clinical application of CO or CORMs. In particular, cardiovascular disease might be the most evident and appropriate choice for the therapeutic use of CO. However, many mechanisms underlying the clinical effects of CO on the cardiac tissue remain unsolved, and sufficient and in-depth research is needed to overcome these obstacles and the limitations of the application of CO or CORMs in cardiovascular disease.
